# Polygenic Risk Scores in Dilated Cardiomyopathy: Towards the Future

**DOI:** 10.1007/s11886-025-02239-2

**Published:** 2025-05-14

**Authors:** Daria R. Kramarenko, Sean J. Jurgens, Yigal M. Pinto, Connie R. Bezzina, Ahmad S. Amin

**Affiliations:** 1 Department of Experimental Cardiology, Amsterdam Cardiovascular Sciences, Heart Failure & Arrhythmias, Amsterdam University Medical Centers, University of Amsterdam, Amsterdam, Netherlands; 2 Cardiovascular Disease Initiative, Broad Institute of MIT and Harvard, Cambridge, MA USA; 3 Cardiovascular Research Center, Massachusetts General Hospital, Boston, MA USA; 4 European Reference Network for Rare, Low Prevalence and Complex Diseases of the Heart (ERN GUARD-Heart), Amsterdam, The Netherlands; 5 Department of Clinical Cardiology, Heart Center, Amsterdam University Medical Centers, University of Amsterdam, Location AMC, Meibergdreef 9, Amsterdam, 1105 AZ The Netherlands

**Keywords:** DCM, GWAS, PGS, PRS, Cardiomyopathy

## Abstract

**Purpose of Review:**

Genome-wide association studies (GWASs) have recently shown that common genetic variations significantly affect the risk of developing dilated cardiomyopathy (DCM). This has enabled the development of polygenic scores (PGSs), which aim to aggregate the impact of multiple common genetic variants across the genome to provide an overall genetic risk score for disease manifestation and disease severity. In this review, we discuss the latest findings pertaining to GWASs and PGSs for DCM and various ways in which PGSs could improve the management of patients with DCM or risk of developing DCM.

**Recent Findings:**

In 2024 the two largest GWAS meta-analyses for DCM were published. Notably, both studies produced PGSs that were able to discriminate healthy subjects from DCM patients which brings promise for potential clinical application of the scores.

**Summary:**

Large-scale GWAS have identified common genetic variants associated with DCM, leading to the development of PGS, which show strong associations with disease risk and hold potential for clinical applications. However, before clinical implementation, further research is needed to explore their utility in real-world settings and across diverse populations.

## Introduction

Dilated cardiomyopathy (DCM) is a myocardial disease affecting approximately 1 in 250–400 individuals. While advancements in diagnostic and treatment strategies have improved disease prognosis and patient survival [1], DCM remains a major cause of heart failure, a leading indication for cardiac transplantation, and is strongly associated with an increased risk of life-threatening arrhythmias [2].

Recent genome-wide association studies (GWASs) and polygenic risk score (PGSs) studies have significantly expanded our understanding of the role of common genetic variants (with a minor allele frequency (MAF) of more than 1%) in DCM, demonstrating their importance alongside rare pathogenic variants (MAF < 0.1%) [3] traditionally associated with disease manifestation and/or severity. In this review, we describe: (1) the current state of DCM diagnostics, (2) advancements in understanding the genetic architecture of DCM, (3) insights obtained through GWAS, and (4) recent progress in analyses of PGSs, including potential clinical applications and associated limitations.

## Improved Understanding of DCM

### Refining Clinical Diagnosis

The diagnosis of DCM is established in patients with a dilated and hypocontractile left ventricle (LV), which cannot be sufficiently explained by other etiologies like ischemia, valvular disease, or hypertension. For isolated cases without a family history of DCM, the diagnosis is primarily based on the clinical manifestation of both LV dilation and systolic dysfunction (although other DCM subtypes with incomplete phenotypic expression have also been proposed) [4]. In contrast, in families with phenotype-positive index cases, the diagnosis in a relative that is found to carry the causative (familial) rare genetic variant may be reached if either LV dilatation or systolic dysfunction are present [5, 6]. 

In 2008, Elliott and colleagues introduced a DCM classification system based on clinical features and etiology, reflecting the growing understanding of the underlying genetic predisposing factors [7]. While they acknowledged the role of genetics in DCM, they ultimately emphasized that clinical manifestations should be central to the classification systems of DCM for better applicability to patient management and treatment decisions. In 2023, the European Society of Cardiology (ESC) updated its cardiomyopathy guidelines and once more highlighted that the diagnostic process in DCM should begin with phenotype-based recognition. This means that symptoms and imaging still play a central role in the diagnostic process and consequently management of patients with DCM. However, once the diagnosis of familial or idiopathic DCM has been established, genetic testing should follow, since the genetic etiology may inform on family screening, long-term prognosis, and clinical decision-making with regard to prophylactic implantation of an implantable cardioverter defibrillator (ICD) to prevent sudden cardiac death from malignant arrhythmias [5]. 

### Advances in Genetics

The traditional view of DCM genetics primarily centers on Mendelian inheritance, wherein rare pathogenic variants in disease-associated genes play a primary role in the development of the clinical phenotype. Based on the systematic analysis of the available genetic and experimental evidence, the ClinGen consortium identified 19 genes with strong evidence to cause DCM (such as *TTN*, *LMNA*, and *BAG3*) [6]. Rare pathogenic variants in these genes are found in about 40% of familial DCM cases and approximately 10% of non-familial (isolated) cases [8, 9]. More recently, large GWASs have started assessing the role of common genetic variation in DCM susceptibility. In the following section, we describe how GWASs and associated PGS studies have increased our knowledge of the genetic architecture of DCM and, particularly, the impact of common genetic variants regarding disease manifestation and disease severity.

## Methods to Study Common Genetic Variants

### Genome-Wide Association Studies

#### General Concept

GWAS aims to identify links between genetic variation and an outcome of interest. To achieve this, genetic variants are statistically tested for association with the outcome, in an unbiased genome-wide manner. While GWAS can be used to study all types of genetic variants including copy-number variants and rarer sequence variations, it typically refers to the assessment of common single nucleotide polymorphisms (SNPs) and small insertion-deletions which are highly prevalent across the human genome (exceeding ten million instances) and which are relatively easy to genotype at scale on genotyping arrays. In line with their relatively high population frequency, common genetic variants generally contribute to phenotypic variability with subtle effects. As such, large cohorts of patients/participants are usually required for a GWAS to detect statistically significant associations between a genetic variant and the phenotype or outcome of interest.

The fundamental idea behind a GWAS is to systematically test millions of variant sites across the genome and assess whether different alleles at each given variant site significantly co-vary with the phenotype of interest. For instance, in the case of a binary disease outcome, the frequency of an allele at a given variant site is compared between individuals with the condition (cases) and those without (controls). Associations of variants with an outcome of interest can be assessed using various statistical tests (e.g., logistic or linear regressions), depending largely on whether the trait of interest is binary (e.g., presence or absence of a disease) or a continuous variable (e.g., diameter ejection fraction of the left ventricle). Additionally, covariates such as sex, genetic ancestry, and year of birth should be included in such statistical models to account for stratification and to prevent confounding [10]. 

#### Overview of the Steps

The first step for any GWAS involves selecting a study population. The study population could be derived from large biorepository datasets (such as UK Biobank [11] or All of Us [12]), or from disease-focused patient cohorts that focus on recruiting disease cases specifically for the study [10].

After the study population is selected, the genetic data needs to be acquired. Most GWASs to date have utilized SNP microarrays, which can typically genotype ~ 200-700k common variations (SNPs) across the genome. Given that microarrays assess only a small fraction of genomic variation, imputation is subsequently used to expand the analysis to non-genotyped SNPs that are in linkage disequilibrium with the genotyped SNPs, thus increasing the number of variant sites studied. This process utilizes ancestry-matched haplotype reference panels and has been highly standardized by means of imputation servers such as the TopMed Imputation Server and the Michigan Imputation Server [13]. More recently, studies have started to utilize whole-genome sequencing (WGS) as a means of genotyping for GWAS approaches. Besides allowing genotyping of both common and rare genetic variants, this approach circumvents the necessity of imputation as it allows for genotyping of variants genome wide.

Before imputation or association analyses are performed, stringent quality control of the genetic data needs to be performed to exclude variant sites and individuals that do not meet predefined criteria [14]. Since GWASs are susceptible to bias from population stratification, quality control also involves crucial steps to infer the genetic ancestry of all samples; subsequent steps may be performed to exclude ancestral outliers. Once the final dataset is of high quality on both phenotypic and genetic levels, the data undergo a genome-wide scan using systematic association analyses, as described above.

#### Output

**Fig. 1:**
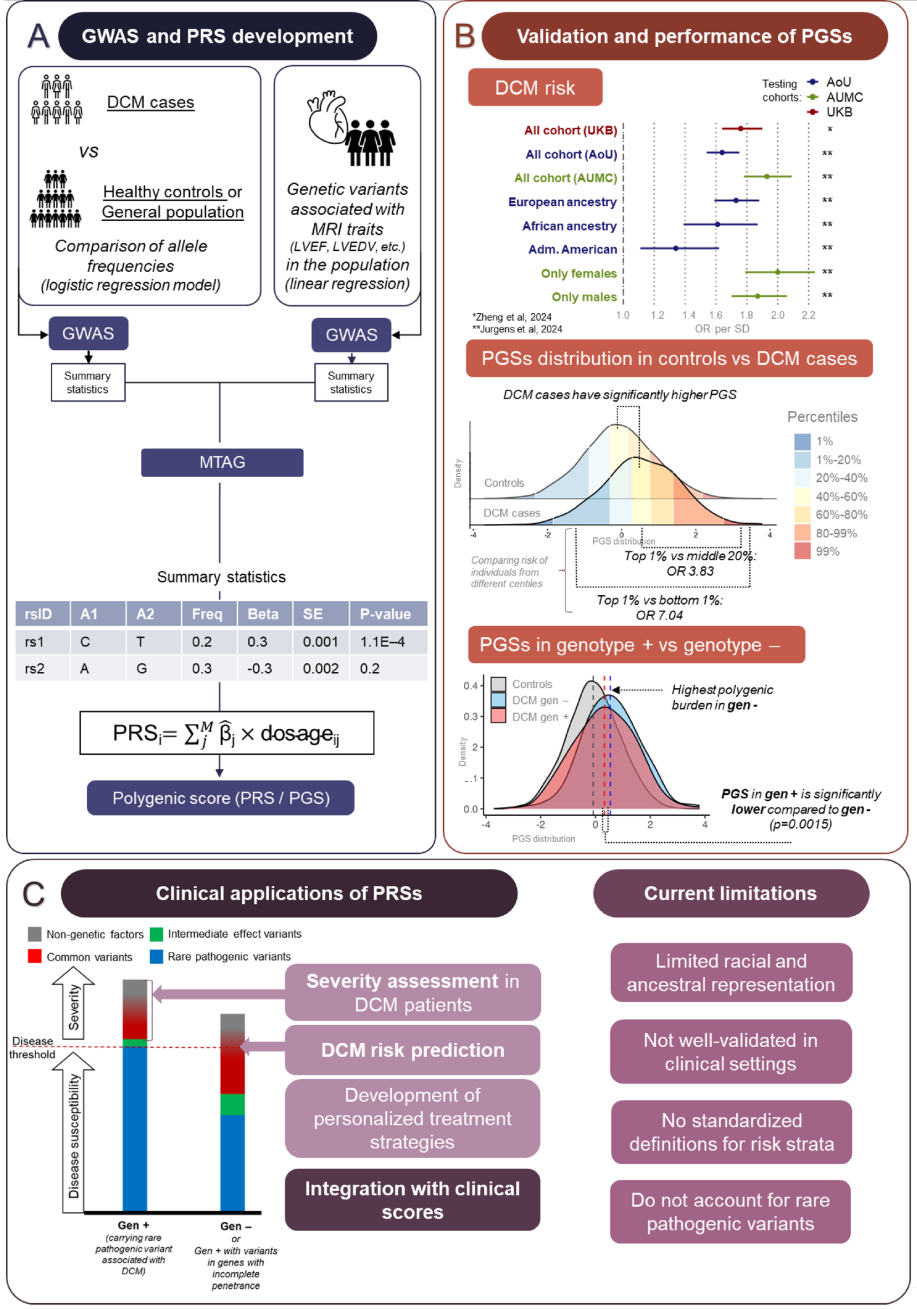
Polygenic scores for dilated cardiomyopathy: development, validation, clinical perspectives, and limitations. **(A)** General overview for development of GWAS and PRS with an example of summary statistics, that is an output of the GWAS and formula used for PRS development. **(B)** PRSs analyses performed by Zheng et al., 2024 and Jurgens et al., 2024. Divided into 3 panels: (1) DCM risk prediction across ancestries and genetic sex; (2) PGS distribution in cases compared to controls; (3) greater polygenic burden in genotype-negative (gen–) versus genotype-positive (gen +) DCM cases. **C**. Left panel: PRS applications in DCM risk prediction, severity assessment, personalized treatment, and integration with clinical scores. Right panel: Current limitations include ancestry bias, lack of clinical validation, no standardized risk thresholds, and exclusion of rare pathogenic variants. DCM: dilated cardiomyopathy, PGS: polygenic score, GWAS: genome-wide association study, MTAG: multi-trait analysis of GWAS, LVEF: left ventricular ejection fraction, LVEDV: left ventricular end-diastolic volume, MRI: magnetic resonance imaging, AoU: All of Us (cohort), AUMC: Amsterdam UMC (cohort), UKB: UK biobank (cohort), OR: odds ratio, SD: standard deviation, gen +: genotype-positive DCM cases (carrying a rare pathogenic variant in DCM causing genes), gen–: genotype-negative DCM cases (rare pathogenic variant was not identified), rsID: reference SNP identifier, A1: effect allele, A2: non-effect allele, Freq: allele frequency, Beta: effect size estimate, SE: standard error, P-value: statistical significance of association

The main results of a GWAS are called summary statistics, which are essentially an overview of all tested variants and their respective effect size, p-value, and other relevant association meta-data (Fig. 1A). Summary statistics are often visualized using a Manhattan plot that displays the p-value for each tested SNP and its association with the phenotype/outcome of interest. In such a plot, the highest points display variants with the most statistically significant association. Summary statistics could subsequently also be processed in downstream analyses such as meta-analysis and multi-trait analysis (MTAG is a method where GWAS from genetically correlated traits are combined to increase statistical power) [15] and used for polygenic scores [10].

**Fig. 2:**
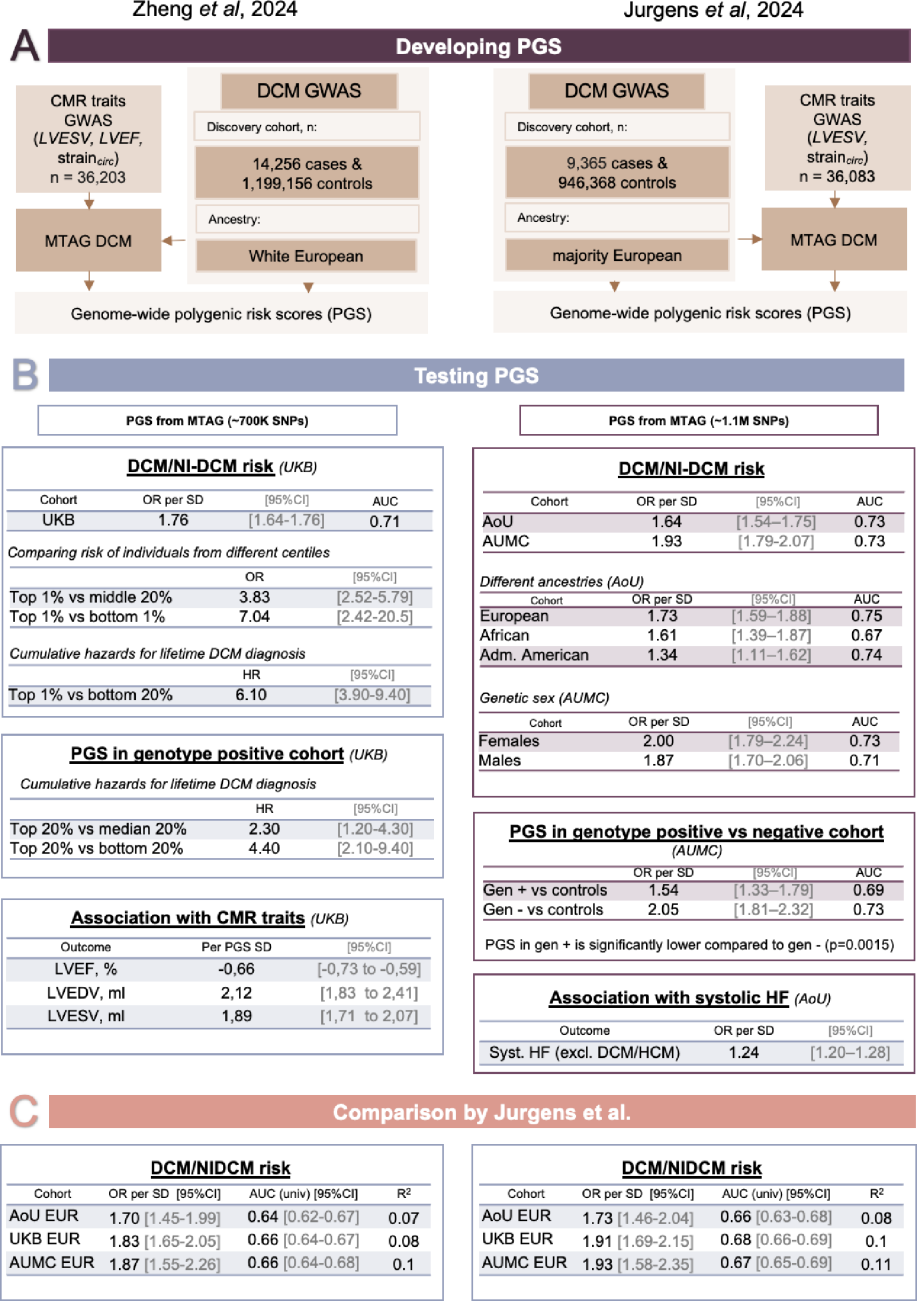
GWASs and PGSs by Zheng *et al*. and Jurgens *et al*. **(A)** Design of GWAS and PGS development. In each study, DCM GWAS was derived through meta-analysis of smaller GWASs conducted in biobank and clinical cohorts for NICM, NI-DCM, and DCM. It was subsequently meta-analyzed with cardiac MRI GWAS traits using MTAG, incorporating key left ventricular phenotypes such as LVEF, LVESV, and myocardial strain. **(B)** DCM PGS associations. The PGSs tested in both studies were constructed out-of-sample, excluding the cohort in which they were evaluated. P-values are two-sided and were calculated from a logistic regression model and not adjusted for multiple testing. All P-values were less than 0.01. Zheng et al.: *Study cohort*: 347,585 unrelated UKB participants with and without DCM. All models included age, age^2^, sex, and first ten genetic PCs as covariates. Jurgens et al.: *AUMC cohort*: Amsterdam UMC cohort with 8,185 participants, of which 978 DCM cases (European ancestry: 7,761 individuals, of which 783 cases​​; Females 4,453 individuals, of which 418 cases​​; Males: 3,732 individuals, of which 560 cases​​; Genotype positive: 193 individuals; Genotype negative: 294 individuals. Model: logistic regression analyses were adjusted sex, and ancestral principal components 1–10. *AoU cohort*: All of Us cohort with 182,701 of which 928 DCM cases (European ancestry only (*N* = 506 cases and 95,510 controls), African ancestry only (*N* = 246 cases and 36,864 controls), and Admixed-American ancestry only (*N* = 107 cases and 28,784 controls)). Model: logistic regression analyses were adjusted for age, age^2^, sex, and ancestral principal components 1–10. (**C**). PGS prediction accuracy comparison within three European ancestry datasets between Jurgens et al. and Zheng et al. DCM PGS. Association results for the PGS constructed from MTAG-DCM with DCM status across three different datasets. Study cohorts: AUMC (DCM cases (*N* = 783), controls: (*N* = 6,978). AoU dataset: samples from Massachusetts General Hospital (MGB) (NI-DCM cases (*N* = 506), controls (*N* = 95,510)). UKB: UK Biobank dataset (NI-DCM cases (*N* = 793), controls (*N* = 325,313)). Model: logistic regression, adjusted for sex, ancestral principal components 1–12, age, age^2^ in UKB and AoU, and only for sex, ancestral principal components 1–12 in AUMC cohort. Data are presented as estimated odds ratios with 95% confidence intervals. R^2^ for each PGS in the respective dataset, where R^2^ represents the residual variance explained by the PGS (computed as the improvement of model R^2^ inclusive of PGS as compared to the model without PGS, divided by the proportion of residual variance); all R^2^ values were computed on the liability-scale to allow better comparisons across datasets. GWAS: genome-wide association study, NI-DCM: nonischemic dilated cardiomyopathy, MTAG: multi-trait analysis of GWAS, OR: odds ratio, 95%CI: 95% confidence interval, SD: standard error, HR: hazard ratio, AUC: area under the receiver operating characteristic curve (AUC provided in part B for models with covariates, AUC in part C, without covariates), R^2^: variance explained, gen +: DCM cases with a rare pathogenic or likely pathogenic rare variant in DCM-causing genes with strong or definitive evidence based on ClinGen curation, gen–: DCM cases without identified rare pathogenic or likely pathogenic rare variant

Given the high number of statistical tests performed in a GWAS, an appropriate statistical significance level is required, with the Bonferroni-corrected p-value threshold typically applied, which equates to *P* < 5 × 10^–8^ (referred to as the genome-wide statistical significance threshold) [16]. The ability to identify SNP associations with a p-value below this threshold rests on the statistical power which in turn depends in part on the size of the (case-control) sample used, the variant effect size, and the frequency of the variants being studied.

### Polygenic Scores Methodology

PGSs are ‘personalized’ scores that are calculated per individual and are based on GWAS summary statistics. Such scores usually represent a weighted sum of the risk alleles that are carried by an individual, with weights derived from the respective effect sizes obtained in GWAS (usually the logarithm of the odds ratio for binary traits, and the beta coefficient for continuous traits) (Fig. 1A). Exactly how variants are included and weighted in PGSs differs widely between different approaches and methods: the selection of risk alleles for the score can be restricted by a certain significance threshold, or the score can be genome-wide, including thousands or even millions of variants [17].

Discussing all available methods is out of scope for the current review, although it must be noted that newer methods tend to only improve prediction marginally compared to the previous gold standard. A crucial component in the evaluation of any PGS, however, pertains to out-of-sample prediction: it is important that the performance of PGS is evaluated in a dataset that is independent of the original GWAS on which it is based (and independent of other data used for the training or tuning of the PGS).

### Early GWASs and PGS Studies in DCM

One of the first common variant association studies for DCM was published by Stark et al. in 2010, comparing 664 DCM cases to 1,874 controls [18]. This study analyzed only 30,920 SNPs and revealed four SNPs displaying association with idiopathic DCM with a p-value below the genome-wide significance threshold (5 × 10^–8^). In this study, only one of these SNPs, namely near the *HSPB7* gene (rs1739843), could be replicated in independent cohorts.

Subsequent GWASs were conducted using genome-wide genotyping arrays followed by imputation, testing a considerably larger number of SNPs spread throughout the genome- [19–28] or exome-based genotyping arrays [29, 30]. The first smaller studies were performed on clinical cohorts, where participants were recruited from (specialized) hospital clinics [19–29]. In 2011, Villard et al., utilizing a discovery cohort comprising 1,179 DCM cases and 1,108 controls, discovered three DCM-associated loci, two of which were replicated in independent samples (rs2234962 located in *BAG3*; rs10927875 located in an intron of *ZBTB17* nearby *HSPB7*) [19]. In 2014, a GWAS by Meder et al. [21] identified a locus near *HCG22* (rs9262636), which was replicated in an independent cohort.

Esslinger et al. were the first to assemble a genotyping dataset of several thousand DCM cases, in 2017. The authors used exome chips to genotype the protein-coding regions in 2796 DCM patients and 6877 controls. They identified previously reported associations near *BAG3* (rs2234962) and *ZBTB17* (rs10927875), as well as novel loci near *TTN* (rs3829746), *SLC39A8* (rs13107325), *MLIP* (rs4712056), *FLNC* (rs2291569), *ALPK3* (rs3803403) and *FHOD3* (rs2303510) [29].

While the above-mentioned GWASs were conducted on participants predominantly of European ancestry, in 2018 Xu et al. published the first DCM GWAS on individuals of African ancestry. They revealed a novel locus in an intron of the *CACNB4* gene (rs150793926). However, they were not able to perform a replication analysis for this SNP [22].

Tadros et al. (2021) performed a meta-analysis of previously published DCM GWAS, and MTAG including LV traits, resulting in 17 DCM-associated loci, 7 of which were novel. While in this study, the concept of PGS was explored in the context of HCM, no DCM-specific PRS was published in this study [26].

Beyond the GWASs that studied risk variants through clinical DCM case recruitment (Table 1A), broader genetic studies leveraging biobank data have since been conducted (Table 1B). These studies leveraged resources such as Biobank Japan [23] and the UK Biobank [23, 25, 30], which comprise atlases of genetic associations, linking phenotypes— such as diseases, biomarkers, and medication usage—to genomic variations through large-scale genome-wide association analyses. While these biobank-based studies included DCM as a phenotype in their analyses, it was not their primary focus. Even though neither of the papers reporting on these large-scale analyses mentioned SNPs associated with DCM, summary statistics for DCM are available in the GWAS Catalog and can be used in meta-analyses with other case-control sets.

The first PGS for DCM was published in 2021 by Garnier et al., based on a GWAS comprising 2651 DCM cases and 4329 controls. The investigators identified and replicated two additional DCM-associated loci in *SLC6A6* (rs62232870) and *SMARCB1* (rs7284877) and confirmed two previously identified DCM loci near *BAG3* and *HSPB7*. Based upon these four SNPs, they constructed several weighted and unweighted PGSs, incorporating between one and eight risk alleles. The weighted PGSs assigned weights to each SNP based on the beta value derived from a sub-meta-analysis of two replication cohorts. They compared the performance of weighted and unweighted PGSs against each other. Each score was composed of a different number of risk alleles (ranging from one to eight), with the five-allele score serving as the reference. The score that included eight risk alleles compared to the reference, demonstrated a three-fold increased risk of DCM, indicating that a higher number of risk alleles correlates with greater disease susceptibility. However, the discriminatory capacity of this score to distinguish between cases and controls remains unclear. Additionally, a limitation of the study was that the PGSs were tested within the discovery cohort, raising concerns about overfitting and generalizability.

Studies identifying genetic loci associated with MRI traits, such as left ventricular volumes and ejection fraction, have also significantly advanced our understanding of DCM genetics. An important study in this regard is the one conducted by Pirruccello et al. [31] Using summary statistics from GWAS on different MRI parameters, the authors constructed several PGSs and assessed their association with the incidence of DCM. Consequently, they demonstrated a strong relationship between a 28-SNP PGS based on SNPs associated with LVESVi, and the occurrence of DCM after adjusting for age, sex, genotyping batch, and the first five principal components of ancestry. [31] Summary statistics of GWAS on MRI traits from this and similar work [25, 32] have since also been used to boost GWAS discovery for DCM through multi-trait analysis (MTAG) [15] frameworks which leverage genetic correlation across different phenotypes (see below) [26–28]. 

### Latest GWASs and PGS Studies in DCM

In 2024, the two largest GWAS meta-analyses studies for DCM to date - Jurgens et al. [28] and Zheng et al. [27] - were published. These studies yielded a substantial boost in genetic discovery for DCM, as they identified several dozen (novel) loci and genes associated with DCM or non-ischemic cardiomyopathy (NI-CM). Both studies conducted meta-analyses of clinical and biobank cohorts and subsequently performed multi-trait analyses, incorporating GWAS for MRI-derived left ventricular (LV) traits (MTAG framework) (Table 1C).

The discovery cohort of Jurgens et al. consisted of 9,365 strict DCM cases and 946,368 controls. Through the GWAS and MTAG, they identified 70 genomic risk loci (38 loci from DCM GWAS and 65 loci from MTAG) at genome-wide statistical significance. The discovered loci showed broad replication in independent samples and were mapped to 63 prioritized genes. The discovery cohort of Zheng et al. consisted of 14,256 DCM/NI-CM cases and 1,199,156 controls [27]. They identified 80 loci (62 loci from DCM GWAS using an FDR threshold of 1% and 54 from DCM MTAG at genome-wide significance) and prioritized 62 putative effector genes. These two studies included approximately 3–4 thousand overlapping cases from 3 cohorts. Additionally, a subset of samples from Zheng et al. were used by Jurgens et al. for replication analyses. A detailed side-by-side comparison of both studies is provided in Fig. 2.

Based on these analyses, several genome-wide PGSs were constructed in both studies, which included hundreds of thousands of genetic variants. In the findings discussed below, we will be referring to the results pertaining to the PGSs that showed the best out-of-sample performance in either study.

Interestingly, Jurgens et al. systematically compared the PGSs from both papers (Fig. 2C) and compared their performance in three datasets of European ancestry (details in Jurgens et al., Supplementary materials). In their analysis, the PGSs based on MTAG summary statistics from both studies showed the best predictive performance. Overall, the authors found that the score by Jurgens et al. performed slightly better, although the confidence intervals still overlapped. Nonetheless, both studies produced PGSs that were able to significantly (but rather weakly) discriminate against healthy controls from DCM patients.

### Perspectives on the Potential Clinical Utility of PGSs

An important aspect of PGSs pertains to their potential clinical utility and the possible clinical scenarios in which these scores could be deployed to improve the current clinical practice with regard to diagnosis, risk stratification, and/or (prophylactic) treatment. Below we describe how PGSs might potentially improve the management of patients with DCM or genetic risk for DCM in different clinical settings (Fig. 1C).

### Individuals at Risk of DCM

One of the most important questions in the evaluation of an asymptomatic patient with an increased risk for DCM is whether the individual is likely to develop disease, and if so, how severe it is expected to become and when it might occur. These questions are particularly relevant for asymptomatic individuals who incidentally have been found to have an increased risk of DCM, for example, those with abnormal ECG findings associated with cardiomyopathy but normal echocardiogram or MRI, those with a family history of DCM where the proband is no longer available for genetic or clinical evaluation, and/or family members of a DCM patient who carry the familial pathogenic variant in a DCM-associated gene.

#### Patients with Possible Risk of DCM as an Incidental Finding

Recent PGSs developed by Jurgens et al. and Zheng et al. have demonstrated the ability to differentiate DCM cases from controls, with DCM odds ratios ranging from 1.5 to 1.9 per standard deviation increase in PGSs and an area under the receiver operating characteristic curve (AUROC) of approximately 0.7 (Fig. 1B) [27, 28]. Individuals with very high PGSs had a markedly elevated risk of DCM, e.g., individuals with a PGSs in the highest 1% had over four- and six-fold higher odds of DCM compared to those with median and low PGSs, respectively. With further development, these scores could serve as a stratification tool to identify individuals at higher risk for DCM, warranting closer clinical evaluation and potential early interventions.

#### Unaffected Family Members with a Family History of DCM

Since recent case-control GWASs include predominantly probands in their study cohorts, they did not evaluate PRS DCM performance for risk stratification in family members. However, previously published 28-SNP PRS for iLVESV [31] demonstrated a significant difference in DCM odds ratios (OR) between clinically affected family members of DCM probands, unaffected relatives, and healthy controls [33]. These data suggest that PRS could be useful to identify family members who may be at increased risk for DCM in situations where the proband is no longer available for genetic evaluation in gene-elusive families where no causative variant has been found.

#### Asymptomatic Carriers of (Familial) Pathogenic Variants

Most carriers of a known pathogenic DCM-causing variant do not develop the disease. For example, in a UK biobank cohort, that includes predominantly White British individuals older than 40 years old, more than 90% of the carriers show no signs or history of DCM [34]. This pattern has also been observed for TTN truncating variants (*TTNtv*), located in cardiac exons. While these variants are the most commonly identified genetic cause of DCM, occurring in 15–30% of genotype-positive cases, they are also present in 0.5% of the general population without DCM [35]. This lack of penetrance of such disease-causing variants suggests that other factors, like common variants, may influence the chance of actually developing DCM. Zheng et al. reported that DCM risk in carriers of DCM-causing variants (predominantly *TTNtv*) was higher compared with gene-negative individuals at the highest PGS centile [27], which is similar to the results of a similar analysis for HCM [36]. Pirruccello et al. addressed this question by testing the 28-SNP LVESVi polygenic score in a cohort of *TTNtv* carriers and showed a positive correlation with LVEDV and LVESV, and a negative correlation with LVEF in individuals without clinical DCM [31]. While follow-up data to assess whether these individuals develop DCM was not available, the observed association between the polygenic score and LV function suggest its potential utility in this setting.

### PRS Application in DCM Patients

In patients with clinical manifestation and an established diagnosis of DCM, PGSs could be of potential value for predicting disease severity and risk for the occurrence of adverse events, such as arrhythmias or heart failure-related outcomes.

#### SCD Risk

Traditional risk prediction models for malignant ventricular arrhythmias, sudden cardiac death, or heart failure in DCM include only clinical factors [37]. In addition, more recently, *ESC Guidelines for the management of cardiomyopathies* highlighted that patients carrying pathogenic variants in high-risk genes (i.e., *PLN*, *DSP*,* LMNA*,* FLNC*,* TMEM43*, and *RBM20*) have significantly increased risk of major arrhythmic events. In line with this, gene-based risk models to predict arrhythmia risk have been developed and incorporated into clinical decision-making algorithms with regard to prophylactic ICD implantation [38]. Nevertheless, it is still unclear, whether common genetic variation could improve the current risk stratification algorithms for primary prevention of SCD. To date, PGSs for DCM were tested only in the context of association with risk of disease development. Recent data suggesting effects of a PRS based on HCM susceptibility variants in modulation of disease severity in HCM underscores the need to explore similar approaches in DCM [36]. 

#### Personalized Therapy

Rare genetic variants have already been linked to left ventricular reverse remodeling (LVRR), which can help to identify DCM patients more likely to experience myocardial recovery and improved cardiac function following pharmacological therapy. For example, *TTN*-related cardiomyopathies have been demonstrated to have a higher likelihood of LVRR compared to other DCM subtypes [39]. In the same manner, common variants may play a role in the response to heart failure therapy and/or antiarrhythmic agents. This concept has been explored in studies of statins, where individuals with the highest polygenic scores received the greatest benefit from treatment [40]. Another potential application for PRS lies in identifying genetic variants associated with adverse drug reactions, as successfully demonstrated in diabetes [41]. These findings highlight the potential of polygenic scores as an enrichment strategy for clinical trials [42] and for guiding personalized treatment, enabling the selection of the most effective therapy for each patient.

#### Combined Scores

However, using PGSs as a stand-alone predictive tool might not be the most effective way to predict risk, considering that recent PGSs explain 7–11% of the variance in DCM susceptibility in the various cohorts (Fig. 2C) [28]. More promising results could be provided using PGSs alongside age, sex, and other traditional clinical risk factors. Similar work has already been done for other diseases, such as atrial fibrillation, coronary artery disease, and diabetes [43]. Notably, additional covariates such as sex, ancestry, and age, already improve score performance. For instance, in such analyses, the area under the curve (AUC) was shown to improve by 0.1–0.2, reaching 0.7–0.75 [28, 43]. Therefore, the clinical value of PGSs will most likely come from their inclusion in established clinical risk models and gene-specific models, provided they are demonstrated to provide predictive information alongside established risk factors [44, 45]. 

To determine whether PGS offers additional prognostic value to established clinical risk models, several statistical methods could be applied. This involves comparing the performance of a baseline model, which includes conventional clinical predictors, to an extended model that additionally incorporates PGS. The choice of statistical model depends on the nature of the outcome: Cox proportional hazards regression is used for time-to-event outcomes, whereas linear or logistic regression is applied for continuous or binary outcomes, respectively.

Formal comparison of model fit is commonly performed using the likelihood ratio test to evaluate whether inclusion of PGS significantly improves model performance. The added predictive value is further assessed through discrimination metrics, such as the (improvement of) concordance index (C-index) or area under the receiver operating characteristic curve (AUC). Reclassification indices and calibration curves may also be useful to assess how PGS might improve/change an existing clinical risk model. To ensure the robustness and generalizability of the findings, model performance should be validated internally—using cross-validation—or externally in independent cohorts.

## Challenges and Limitations

### Clinical Translation

PGSs have not yet been sufficiently studied in real-world clinical settings, which limits their applicability. Even though PGSs scoring files are now publicly available, allowing for their calculation in individual patients, it remains unclear how to interpret these values in terms of individual risk. Although we have a better understanding of the polygenic burden and its associations with MRI-derived traits, the key question remains where the thresholds should be set to categorize individuals as high or low risk?

### Accounting for Rare Pathogenic Variants

PGS, as a sum of the effects of common variants (with MAF > 0.05-1%), usually does not incorporate the effects of rare genetic variants. Jurgens et al., demonstrated that even though the contribution of common genetic variants is higher in genotype-negative individuals, their role is also significant in rare pathogenic variant carriers. This might affect disease severity in DCM patients or the risk of DCM manifestation for variants with incomplete penetrance (Fig. 2B) [28]. One of the approaches to address this issue could be to combine monogenic and polygenic risks, which have been explored for breast cancer risk [46]. 

### Ethical Considerations

PGSs depend on the power and quality of the underlying GWAS. Most GWASs to date lack diversity and PGSs are generated and tested in populations of predominantly European ancestry. This lack of diversity limits the applicability of PGSs to individuals of non-European descent, raising challenges concerning equity and transferability [47, 48]. Unfortunately, the same remains true for DCM PGSs. Even though Jurgens et al. tested polygenic scores in cohorts with African and Admixed-American ancestry, the authors observed that odds ratios were lower compared to those in the European cohort (1.61 for African, 1.34 for Admixed-American, and 1.73 for European) (Fig. 1B). Notably, only one of the previously published GWAS was performed in a cohort of African ancestry [22]. Addressing this gap will require large-scale GWAS in diverse populations, as well as trans-ancestry meta-analyses and international collaborations aimed at improving the accuracy, equity, and clinical applicability of PGSs globally. This have been extensively discussed in the recent review by Kachuri et al. [49]

## Conclusions

Recent studies have utilized large GWAS approaches to identify common genetic variants associated with DCM. These studies have subsequently built PGSs that demonstrate strong associations with DCM risk, suggesting a potential application for these scores in clinical practice. However, before PGSs can be clinically utilized, further research is needed to ensure their applicability to specific real-world clinical scenarios and efficiency among populations with diverse ancestries. The added value of PGSs should also be demonstrated in combination with existing clinical prediction tools, similar to other diseases where PGSs have significantly improved predictive accuracy and patient reclassification. This could lead to more precise risk stratification, potentially benefiting both the general population and specific high-risk cohorts. Clearly, progress in this domain will necessitate (inter)national collaboration to allow the establishment of large patient databases with detailed well-defined clinical data to enable well-powered association studies.

### Key References

- Zheng SL, Henry A, Cannie D, Lee M, Miller D, McGurk KA, et al. Genome-wide association analysis provides insights into the molecular etiology of dilated cardiomyopathy. Nature Genetics. 2024; 1–13.
  - Findings from this study suggest that DCM PRS is significantly associated with DCM in the general population and modify penetrance in carriers of rare DCM variants.
- Jurgens SJ, Rämö JT, Kramarenko DR, Wijdeveld LFJM, Haas J, Chaffin MD, et al. Genome-wide association study reveals mechanisms underlying dilated cardiomyopathy and myocardial resilience. Nature Genetics. 2024; 1–10.
  - Findings from this study suggest that DCM PRS is significantly associated with DCM across different ancestry groups, show differing contributions to DCM depending on rare pathogenic variant status and associate with systolic heart failure across various clinical settings.

**Table 1:** Overview of key genome-wide association studies (GWAS) of dilated cardiomyopathy (DCM)

Reference	Cohorts (cases/controls)	Number of identified loci associated with DCM	MTAG	PGS
(A) **Studies in clinical cohorts**				
Stark et al., 2010 [18]	Discovery: 664/1,874* Replication: 1,246/1,756*	4 loci identified (Replicated: 1 locus in *HSPB7* (rs1739843))	-	-
Villard et al., 2011 [19]	Discovery: 1,179/1,108* Replication: 1,165 /1,302*	3 loci identified (Replicated: 2 loci: in *BAG3* (rs2234962), an intron of *ZBTB17* nearby *HSPB7* (rs10927875))	-	-
Meder et al., 2014 [21]	Discovery: 909/2,120* Replication: 3,234/5,590*	1 locus identified (Replicated: 1 locus near *HCG22* (rs9262636))	-	-
Esslinger et al., 2017 [29]	Discovery: 2,796/6,877* Replication: -	8 loci identified: near *BAG3* (rs2234962), *ZBTB17* (rs10927875), *TTN* (rs3829746), *SLC39A8* (rs13107325), *MLIP* (rs4712056), *FLNC* (rs2291569), *ALPK3* (rs3803403) and *FHOD3* (rs2303510)	-	-
Xu et al., 2018 [22]	Discovery: 662/1,138 (African American or Afro-Caribbean ancestry) Replication: -	1 locus identified in an intron of *CACNB4* (rs150793926)	-	-
Garnier et al., 2021 [24]	Discovery: 2,651/4,329* Replication: 584 /963*	5 loci identified (Replicated: 4 loci: in *BAG3* (rs61869036), *HSPB7* (rs10927886), downstream *LSM3* (rs62232870), in *SMARCB1* (rs7284877)	-	Performed but not available for use.
Tadros et al., 2021 [26]	Discovery: 5,521/397,323* Replication: -	13 loci identified	+	Available only for HCM
(B) **Biobank studies**				
Backman et al., 2021 [30]	Discovery: 858/387,072* Replication: 748/94,883*	N/A	-	-
Sakaue et al., 2021 [23]	Discovery: 1,444/353,937 Europeans; 417/177,745 East Asians Replication: -	N/A	-	-
Ning et al., 2023 [25]	Discovery: 1,303/5,281* Replication: -	N/A	-	-
(C) **Combined clinical and biobank cohorts**				
Zheng et al., 2024 [27]	Discovery: 14,255/1,199,156 controls* - DCMNarrow: 6,001 cases* - DCMBroad: 9,299 cases* MTAG + 36,203 participants* Replication: -	80 genetic susceptibility loci (FDR < 1%) and 61 prioritized putative effector genes	+	A detailed side-by-side comparison of PGSs from both studies is provided in Fig. 2
Jurgens et al., 2024 [28]	Discovery: DCM: 9,365/946,368* NICM: 13,478/932,873* MTAG + 36,083 Replication: 13,258 /1,435,287*	70 genome-wide significant loci, which show broad replication in independent samples and map to 63 prioritized genes.	+	

The table summarizes major GWAS investigating genetic susceptibility loci for DCM. Columns include: (i) the reference, study, and year of publication; (ii) the number of cases and controls included in discovery and replication cohorts, with an asterisk (*) indicating studies conducted predominantly in individuals of European ancestry; (iii) the number of loci identified as significantly associated with DCM, including the number of loci that were replicated, if applicable; (iv) whether multi-trait analysis of GWAS (MTAG) was performed; and (v) whether polygenic scores (PGS) were constructed and tested in the respective study

Summary-level data for some studies are available across several public repositories. The GWAS Catalog provides access to GWAS summary statistics (EFO ID: EFO_0000407, https://www.ebi.ac.uk/gwas/efotraits/EFO_0000407*)*, while the PGS Catalog contains polygenic scores (https://www.pgscatalog.org/trait/EFO_0000407/*).* The Cardiovascular Disease Knowledge Portal (https://cvd.hugeamp.org/downloads.html*)* offers both GWAS summary statistics and corresponding PGS data (Availability of these datasets was confirmed on 04 January 2024).

DCM: dilated cardiomyopathy, PGS: polygenic score, GWAS: genome-wide association study, MTAG: multi-trait analysis of GWASs

## Data Availability

No datasets were generated or analysed during the current study.
